# Investigating management choices for canine heartworm disease in northern Mississippi

**DOI:** 10.1186/s13071-017-2450-8

**Published:** 2017-11-09

**Authors:** Tobi N. Ku

**Affiliations:** College of Veterinary Medicine, Mississippi State University, 240 Wise Center Drive, Starkville, MS USA

**Keywords:** Heartworm disease, Heartworm preventive, Macrocyclic lactone, *Dirofilaria immitis*

## Abstract

**Background:**

There are concerns that the chronic use of macrocyclic lactone preventives to kill adult heartworms (“soft-” or “slow-kill”) may have contributed to the development of macrocyclic lactone resistance. This prospective analysis was designed to expand our understanding of currently employed treatment decisions, protocols utilized in a “slow-kill” methodology, and trends in heartworm prevention in a region with concerns about macrocyclic lactone resistance. We tested the hypothesis that practitioners underestimate the actual percentage of heartworm-positive dogs treated with “slow-kill” therapy. Owners’ financial concerns and veterinarians’ efforts at meeting client preferences are the primary reasons for employment of “soft-kill” treatment.

**Methods:**

A prospective analysis of dogs determined to be heartworm-positive when presented to a mixed-animal practice in northern Mississippi was conducted for the second quarter of 2016. Client records were scrutinized for heartworm preventive purchase history. Veterinarians in the four-doctor practice completed a questionnaire regarding their beliefs and practices of heartworm treatment.

**Results:**

Forty of 321 canine patients tested heartworm-positive with a commercial antigen test kit. Of these, two were considered to be due to possible product failure. The majority (75.0%) of patients received a “slow-kill” method, a percentage greater than that estimated by the practitioners. Patients were equally likely to have received adulticidal treatment as they were to receive no treatment (12.5%). Injectable moxidectin was the most common preventive used in “slow-kill” treatment (80.65%). Client financial concerns were cited as the primary reason for choosing “slow-kill” treatment (79.0%).

**Conclusions:**

Despite American Heartworm Society recommendations, clients and veterinarians prefer the “slow-kill” method of heartworm treatment. Trends in patient heartworm preventive history show that poor client compliance remains the predominant explanation for heartworm infection. Thus, consistent use of existing, effective heartworm preventives should be the primary goal in reducing prevalence of heartworm infection, regardless of the recognized threat of resistance. It is also noteworthy that practitioner estimates may be suspect in their accuracy. Further study is needed on the risks and efficacy of “slow-kill” treatment and the effects of different ML preventives for this off-label use.

## Background

Macrocyclic lactone (ML) preventives, used for heartworm (HW) disease prophylaxis, are under threat of resistance by their target organism, *Dirofilaria immitis*. The Food and Drug Administration, Center for Veterinary Medicine saw a dramatic increase in lack-of-efficacy (LOE) claims in the beginning of the twenty-first century, especially in endemic areas of the southeast United States [1]. Additionally, a 2014 survey found that 74% of all responding Louisiana veterinary practices had seen at least one LOE case within the previous year [2]; and preliminary results from currently unpublished, ongoing questionnaires throughout the Mississippi Delta region (Alabama, Arkansas, Mississippi, Missouri, Louisiana and Tennessee) seem to follow this trend [3]. Microfilariae from certain *D. immitis* strains have persisted through ML treatment, previously known to clear this stage of the parasite [3]. During the development of new ML preventive combinations, it was found that previously effective molecules no longer demonstrated 100% efficacy against a certain HW isolate (MP3) [4]. In recent years, other research teams have successfully isolated ML-resistant *D. immitis* strains from cases of LOE [2, 4]. Thus, it is well accepted that *some* heartworm isolates are resistant to the effects of ML preventives.

HW resistance to ML preventives is a topic of concern, particularly in the Mississippi Delta and bordering regions, as evidenced by increased LOE claims in the area. It has been suggested that certain therapeutic practices, such as the off-label incorporation of ML for “slow-kill” adulticidal therapy in treating heartworm infections (HWI), contribute to the development of resistance [4]. This practice is not currently recommended by the American Heartworm Society (AHS) for this and other reasons. In light of these concerns, HW disease management and decision-making processes of both clients and veterinarians are of interest. Retrospective analyses of medical records have been used as important tools to observe owner compliance and patient histories in regards to HW disease and prevention [2, 5]. However, there are limitations in performing retrospective analyses for the purpose of understanding veterinarian and pet owner behavior.

First, many practices lack the time, appropriate software, or interest to record the clinical justifications for each treatment decision consistently and accurately. As a result, studies rely on practitioner self-reporting by memory, and thereby have the potential to contain error or personal bias. In one study, even when owners and veterinarians believed that a patient had received HW preventive with “perfect” compliance, gaps of coverage were still detected in a high number of cases [5]. A recent presentation of preliminary data from HW management questionnaires in the Mississippi Delta emphasized not only the need to bridge academic and clinical environments in HW treatment, but also mentioned the possible effects of clinician opinions and biases on questionnaire analysis results [3].

In order to better understand treatment decisions for HW-positive patients, protocols utilized when following a “slow-kill” method, and trends in HW prevention history, this study was designed to analyze prospectively the treatments used to manage HW-positive dogs in the MS Delta. We also sought to determine whether practitioner estimates for the prevalence of “slow-kill” therapy in this clinic differed from the actual numbers detected through scrutiny of client purchase and patient medical records.

## Methods

### Participants

This study was performed at a mixed-animal private practice in Oxford, Mississippi, employing four veterinarians. Oxford falls within the region of high density for LOE claims [1]. From April to June 2016, canine patients were tested for HWI using the SNAP® Heartworm RT or SNAP^®^ 4Dx® Plus Test (IDEXX Laboratories, Westbrook, Maine, USA) during routine annual examinations or when experiencing suspect clinical signs, at a doctor’s discretion. The SNAP tests, like all HW antigen tests, can indicate HWI as early as 6 months after transmission. Dogs testing positive for HWI were entered into the study.

### Data collection

For HW-positive cases, client transaction records and patient charts were carefully scrutinized for HW preventive purchase gaps, purchases for multiple patients in the same household, and patient HW testing and/or treatment history. Prevention purchases for multiple patients in the same household were included in the criteria because product sharing may indicate possible compromises in HW protection [5]. When available, heartworm preventive history was studied from 2 years before positive testing, or from birth if the patient was less than 2 years old. Patients were assigned a status under the following criteria:

- Consistent – no gaps in coverage greater than 3 months; such gaps in coverage may be covered by product “reach-back” [6].
- Inconsistent – gaps in coverage greater than 3 months.
- None – no record of preventive use.
- Unknown – patients without available medical records for the period of interest (eg, newly adopted pets).

These treatment protocols were recorded in one of three categories: adulticidal therapy, indicating the administration of melarsomine (Immiticide®, Merial Limited, Duluth, Georgia); “slow-kill” method, indicating the off-label use of an ML preventive as a HW adulticide, in addition to at least 1 month of oral doxycycline; and no treatment. The type of ML preventive used for “slow-kill” therapy was also recorded in each case.

### Practitioner and client opinions

Veterinarians were asked to complete a questionnaire (see table) regarding their methods and beliefs with respect to HW treatment protocols. They were asked to estimate the percentage of HW-positive dogs diagnosed under their care receiving “slow-kill” therapy. From their answers we determined the average number of HW-positive dogs in this practice that receive “slow-kill” therapy. Practitioners reported whether they began discussions with clients regarding HW treatment by introducing arsenical therapy or “slow-kill” therapy. They were also asked to indicate and rank the primary factors that they believe influence clients to choose “slow-kill” over arsenical HW treatment, and to expand on these reasons if possible.

### Data sorting and analysis

This practice utilized both digital and physical (paper) medical records, so all records were pulled from AVImark® software (Logistic, 2009) or physical patient files and analyzed using Microsoft® Excel (Microsoft Office, 2016). Window-of-infection (WOI) analyses were performed for patients with a consistent history of HW preventive use in the previous 2 years using the Merial© “window of infection” program (http://www.heartwormwoi.com/). The window of infection is defined as the period of time in which the current infection is most likely to have occurred. This time period starts at 9 months prior to the last negative HW test and ends 6 months before the positive HW test. Purchase gaps of 45 days or more within the WOI indicate compliance failure and argue against product failure [5].

## Results

### Heartworm prevention histories

**Fig. 1:**
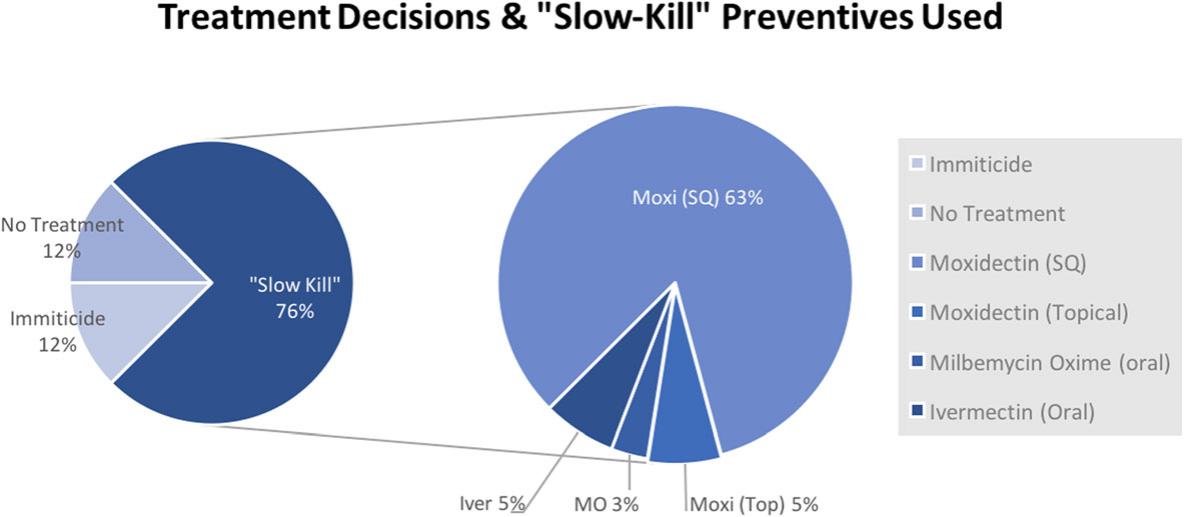
Heartworm treatment decisions and macrocyclic lactone preventives chosen for “slow-kill” treatment

**Fig. 2:**
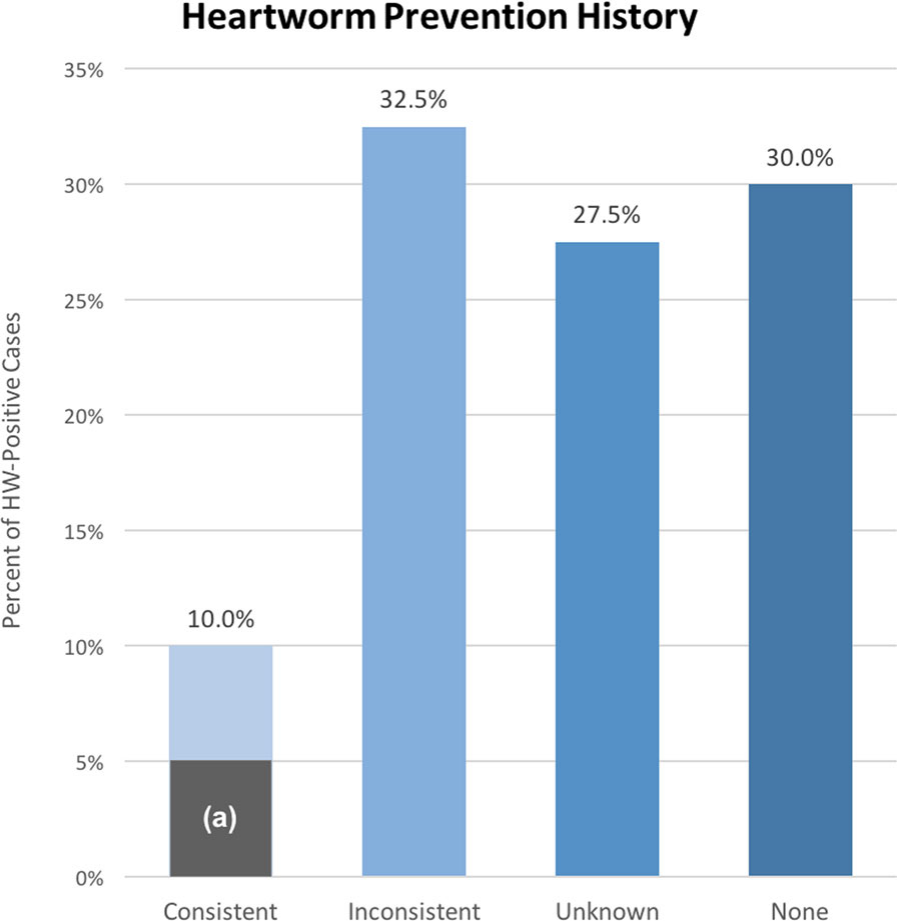
Prevention history for heartworm-positive patients. Columns represent different levels of heartworm prevention consistency determined by analyzing purchase history 2 years prior to heartworm antigen-positive test. The shaded region (**a**) in the first column denotes the proportion of cases that was not found to have purchase gaps >45 days after WOI analysis (5.0%)

**Fig. 3:**
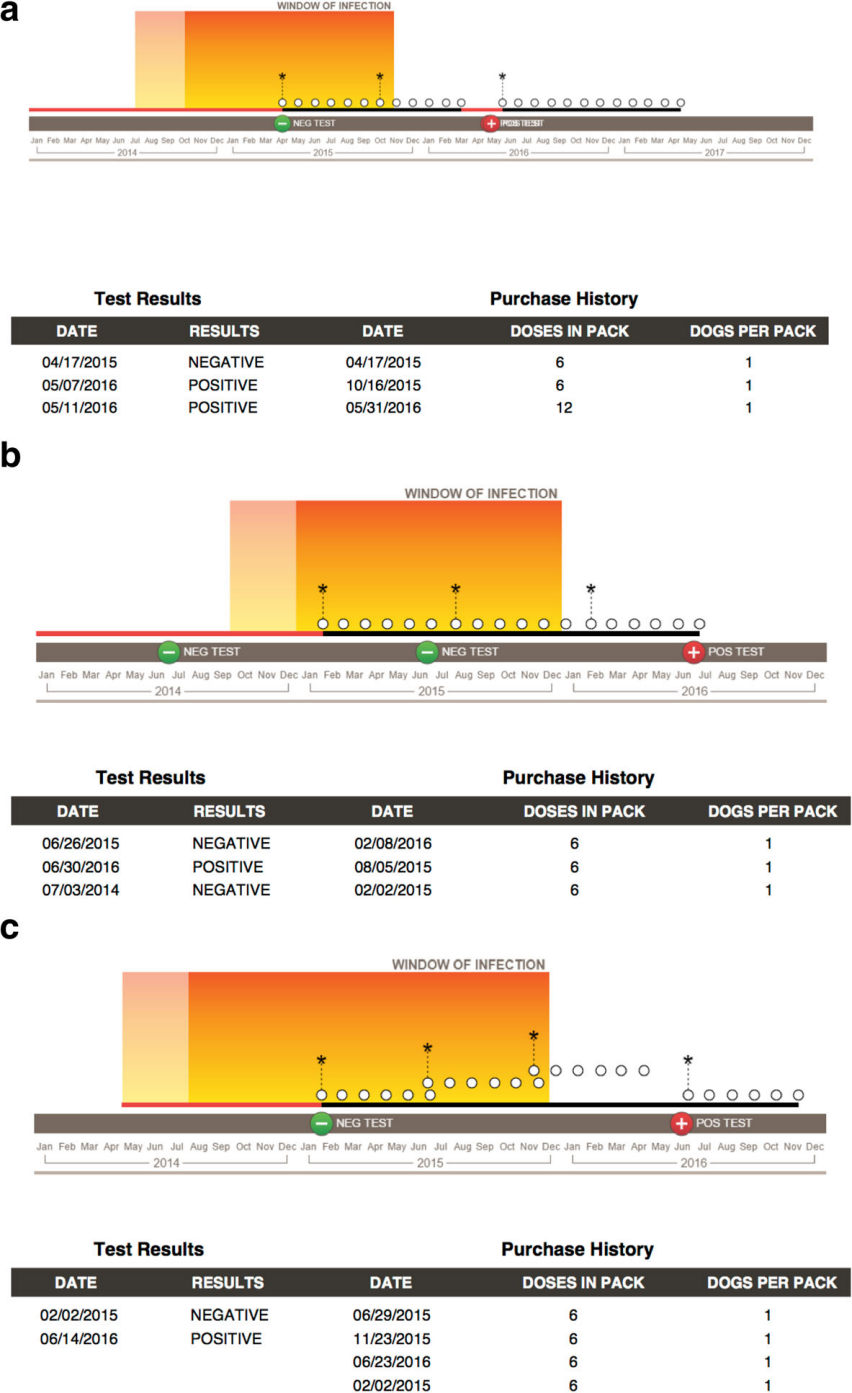
Window of infection analyses for three heartworm-positive patients. The window of infection (WOI) is the period of time in which the current infection is most likely to have occurred and includes the period of time from 9 months prior to the last negative heartworm test through the date 6 months prior to the positive heartworm test. The finding of purchase gaps 45 days or greater within the window of infection argue against product failure and, instead, indicate compliance failure. In these diagrams, a white circle represents a single heartworm preventive dose, an asterisk indicates the purchase of additional preventive medication, and a positive or negative symbol denotes the time and result of heartworm testing for the patient in question. These dogs (**a**, **b**, **c**) appeared to have consistent heartworm prevention coverage during initial scrutiny of patient records, but gaps >45 days were apparent in the WOI analyses. These gaps are symbolized by red coloration of the horizontal timeline. It is of interest that all three dogs showed good compliance after the first purchase of medication but were infected prior to having received heartworm prophylaxis

Of 321 patients tested for HW over the period of this study, a total of 40 patients tested HW-positive (Fig. 1). Client records revealed that over half of all HW-positive patients had inconsistent history (32.5%) or no history (30.0%) of HW prevention in the previous 2 years (Fig. 2). The remaining cases consisted largely of patients with unknown HW prevention histories (27.5%). Few patients had consistent HW preventive coverage (10.0%); one such patient had previously tested positive and was currently under slow-kill treatment. Window-of-infection (WOI) analyses were performed for the remaining three patients that appeared to have a consistent preventive history. These analyses identified preventive purchase gaps of over 45 days within the WOI for all three cases under consideration (Fig. 3). There were only two LOE claims submitted to pharmaceutical companies for compensation during this period.

### Heartworm treatment methods

Of the HW-positive patients included in this study, a majority received treatment using a “slow-kill” method (75.0%; Fig. 1). Patients were equally likely to receive an arsenical (melarsomine) as they were to be given no treatment (12.50%). Of the five patients who received no treatment, two were experiencing severe health complications and were euthanized before HW treatment. Another was in the care of a rescue group and was transferred to a different organization before HW treatment was considered. The remaining two patients received no HW treatment based on client choice.

Four different HW preventive choices were utilized for “slow-kill” treatment in this practice: injectable moxidectin (ProHeart® 6, Pfizer Inc., Madison, New Jersey), topical moxidectin/imidacloprid (Advantage Multi®, Bayer Animal Health, Shawnee, Kansas), oral milbemycin oxime/spinosad (Trifexis®, Elanco Animal Health, Greenfield, Indiana), and oral ivermectin/pyrantel (Heartgard® Plus, Merial Inc., Duluth, Georgia). Of these products, a majority (83.33%) of cases were treated with injectable moxidectin. Oral ivermectin (6.67%), milbemycin oxime (6.67%), and topical moxidectin (3.33%) were chosen far less in “slow-kill.”

### Treatment methods questionnaire

Practitioner questionnaire results are depicted in the table. When asked to estimate the treatment decisions regarding their HW-positive canine patients, these veterinarians believed, on average, that 53.75% of HW-positive dogs in this practice received slow-kill therapy (instead of adulticidal treatment or no treatment); but these estimates ranged from 10.0% to 75.0%,with a median of 65.0% (see Table 1, part a). All practitioners reported that, when discussing HW treatment options with clients, they introduced adulticidal therapy before “slow-kill” therapy options (see Table 1, part b).

**Table 1:** Practitioner questionnaire and results

Survey questions		Individual responses				Average
		A	B	C	D	
(a) Estimate the percentage of dogs diagnosed with HWI that receive slow/soft-kill therapy in this practice under your care.		10%	75%	60%	70%	53.75%
(b) When discussing protocols for heartworm treatment with clients, which adulticide method do you usually begin your discussion with?	Macrocyclic lactone	0%	0%	0%	0%	0%
	Melarsomine	100%	100%	100%	100%	100%
(c) Why do clients choose “slow-kill”? Please indicate the relative importance of each factor.	Owner’s convenience	0%	35%	7%		14%
	Arsenical concern	0%	10%	6%		2.33%
	Advanced patient age	0%	20%	10%		4%
	Concurrent life-threatening disease	0%	20%	2%		1.33%
	Cost concerns	100%	60%	75%		78.33%

One practitioner did not complete the questionnaire portion shown in the table, part c. Results from this portion were obtained from three practitioners only. The practitioners cited client financial concerns as the primary deciding factor for clients who chose “slow-kill” therapy (78.3%), since adulticidal therapy requires additional charges for the drug, drug administration and hospitalization. The second-most commonly cited factor was convenience (14.0%), as many clients reportedly disliked the confinement aspect of adulticidal therapy, particularly in cases involving active, asymptomatic patients. Patient age was another influential factor in HW treatment considerations, as clients often mentioned disliking stressful or expensive treatment procedures for older pets. Arsenical concern (2.33%) and preexisting life-threatening disease (2.33%) were less commonly cited reasons for choosing “slow-kill.”

## Discussion

Herein, we report on preventive protocols in HW-infected dogs, veterinarian and client HW management decision-making, and HW treatment protocols in a Mississippi Delta clinic. We also tested the hypothesis that: “*practitioners underestimate the percentage of HW-positive cases treated with “slow-kill” therapy.*”

Heartworm infection in this practice resulted most often from inadequate (32.5%), unknown (27.5%) or absent (30.0%) ML preventive use. Even in the 10% of HW-positive dogs that consistently received ML, deeper analysis revealed purchase gaps of 45 days or greater in three of four cases. Thus, 95% of HW-positive patients in this study had inadequate HW protection. Furthermore, HW resistance to ML (based on paid LOE claims) comprised only two (0.62%) of the 321 dogs tested during this time period. This is compatible with previous observations from a retroactive case study exploring the factors that may have contributed to an increase in LOE, showing that, over the past 10 years, annual LOE cases made up ≤1.3% of total HW tests performed [7].

Our results show that in this practice, HW-positive cases were overwhelmingly treated with “slow-kill” therapy during the months of this study. These data differ from preliminary results of regional questionnaires in which practitioners reported slow-kill therapy usage in less than 10% of HW-positive cases [3]. The in-clinic survey results from this study (Table 1a) show that, although these practitioners have higher estimates than other veterinarians in the Mississippi Delta region, they still underestimate the use of “slow-kill” therapy in their own practice (with a percent error of 28.3% between mean estimate and actual value).

The results of this comparison, along with the generally low return rate on surveys, underscore concerns that practitioner questionnaires may be inaccurate tools for such data collection. However, one of the major limitations of this study is its small scope, as it enrolled only a single practice with four veterinarians and analyzed only 3 months’ activity, which is not a representative sample of veterinary practices. Thus, the results of this study cannot be generalized to other practices in the encompassing region. In reality, such extensive review of medical records is often impractical for the majority of veterinary practitioners. This is due to time constraints, digital medical recording practices that are not designed for retrieving such information, and paper medical records that are cumbersome and time-consuming to search through. Perhaps most significant is the fact that many medical records retain inadequate data to answer the questions of research. Thus, while small case studies like this one can provide a unique, deeper insight into HW disease management, self-report questionnaires, despite their flaws, remain an important and efficient tool for investigating and understanding circumstances in veterinary practices.

The ML preventive that was chosen for use in a majority of “slow-kill” treatments in our study was injectable moxidectin. This appears to be due to this preventive’s decreased dependence on client compliance, the fact that it is administered in the practice by a veterinary professional, and that it requires administration only every 6 months.

The questionnaire results in this study reveal that clients who elect slow-kill therapy for their HW-positive dogs are primarily influenced by financial factors. While this finding represents an important consideration for practitioners, it also calls for awareness of the financial environment pervading the Mississippi Delta region – that is, a significant percentage of clients in this highly HW-endemic region experience severe poverty. According to US Census data from 2010 to 2014, the county that the practice in this study serves has a median annual household income ($41,343) much lower than that of the United States overall ($53,482) and a poverty level (26.1%) higher than that of the United States (15.6%). In fact, many states included in the Mississippi Delta region have low household incomes and high poverty levels compared to the national average [8]. The results of this study support the fact that economic factors play a significant role in HW management decisions and should be taken into consideration. Therefore, it is imperative that we explore less expensive alternatives of HW treatment using melarsomine; more rapid forms of “slow-kill” utilizing ML preventives and doxycycline; or possibly as-yet undefined combinations of these agents.

## Conclusions

This study provides a perspective on the prevalence of “slow-kill” therapy in a Mississippi Delta practice, demonstrating its heavy employment; suggests reasons for this decision on behalf of both veterinarians and clients; and shows that this methodology is, in fact, used more often than estimated by doctors in the practice, despite AHS recommendations. Practitioner questionnaires reveal client financial concerns as the primary factor driving HW treatment decisions, although convenience is also shown to be an important factor. Injectable moxidectin is the ML preventive of choice for “slow-kill” therapy in an overwhelming number of cases included in this study. As cost and convenience levels of current melarsomine adulticidal procedures are high, the author advocates for research into less expensive alternatives. Research exploring the efficacy of injectable moxidectin should be a particular priority in light of its prevalence in this study, the benefits of its administration method, and recent studies regarding the efficacy of topical moxidectin as an adulticide.

Findings from medical records do not reflect practitioner estimates of “slow-kill” use in this practice, with “slow-kill” use being far more prevalent than estimated. This should be taken into consideration when self-report questionnaire studies are designed. While case studies such as this one can serve as useful tools, development of software allowing more efficient and accurate data collection from multiple practices could prove to be extremely useful in prospectively designed epidemiologic studies of private-sector veterinary practices.

Ultimately the data, in addition to WOI analyses, suggest that poor client compliance with HW preventive administration remains the predominant cause of HW infection. This suggests that compliance with existing, effective HW preventives remains the primary factor in reducing HW disease prevalence, regardless of the recognition of resistance.
